# Supplementary material for: Characterization of a PERK Kinase Inhibitor with Anti-Myeloma Activity
Source: Cancers (Basel). 2020 Oct 5;12(10):2864. doi: 10.3390/cancers12102864 (PMC7601861; doi:10.3390/cancers12102864)
Supplement: Supplementary file 1 [file cancers-12-02864-s001.pdf]

Supplementary Materials: Characterization of a PERK Kinase Inhibitor with Anti-Myeloma Activity

Tina Bagratuni, Dimitrios Patseas, Nefeli Mavrianou-Koutsoukou, Christine Ivy Liacos, Aimilia D. Sklirou, Pantelis Rousakis, Maria Gavriatopoulou, Evangelos Terpos, Ourania E Tsitsilonis, Ioannis P. Trougakos, Efstathios Kastritis and Meletios A. Dimopoulos

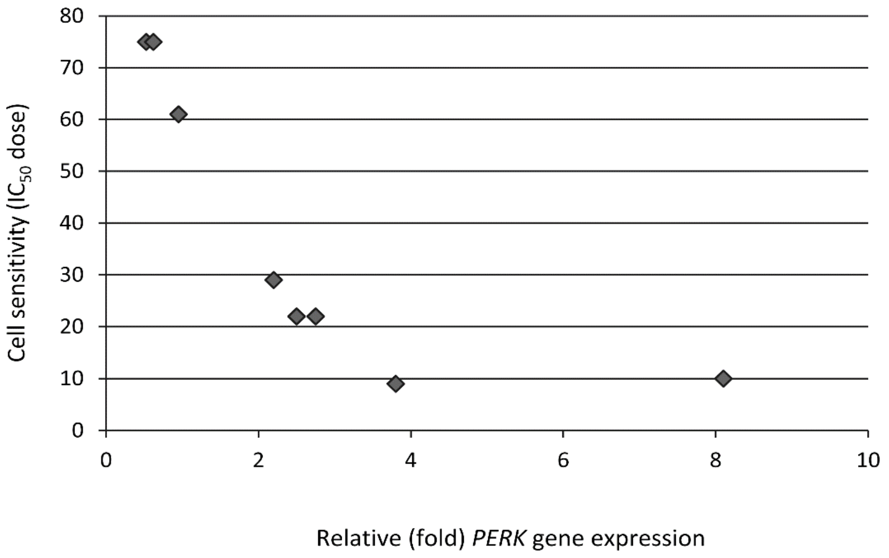

Figure S1. Correlation of PERK expression levels with sensitivity to PERK inhibition in all cell lines.

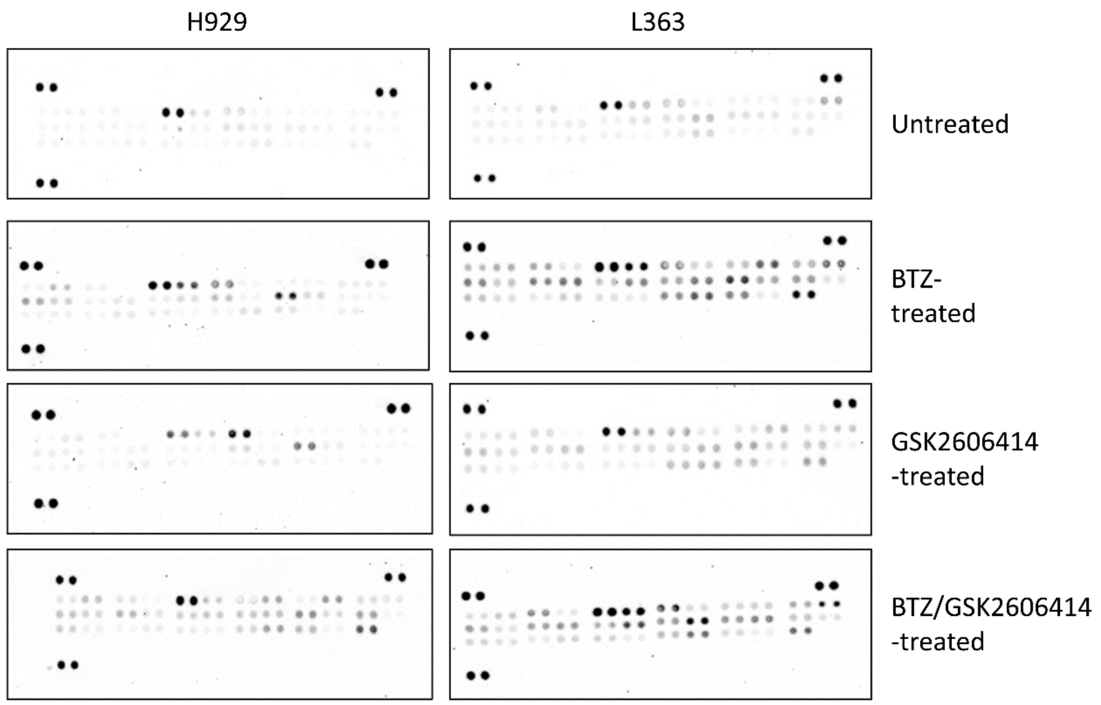

Figure S2. Apoptosis related proteome profiler in H929 and L363 cell lines.

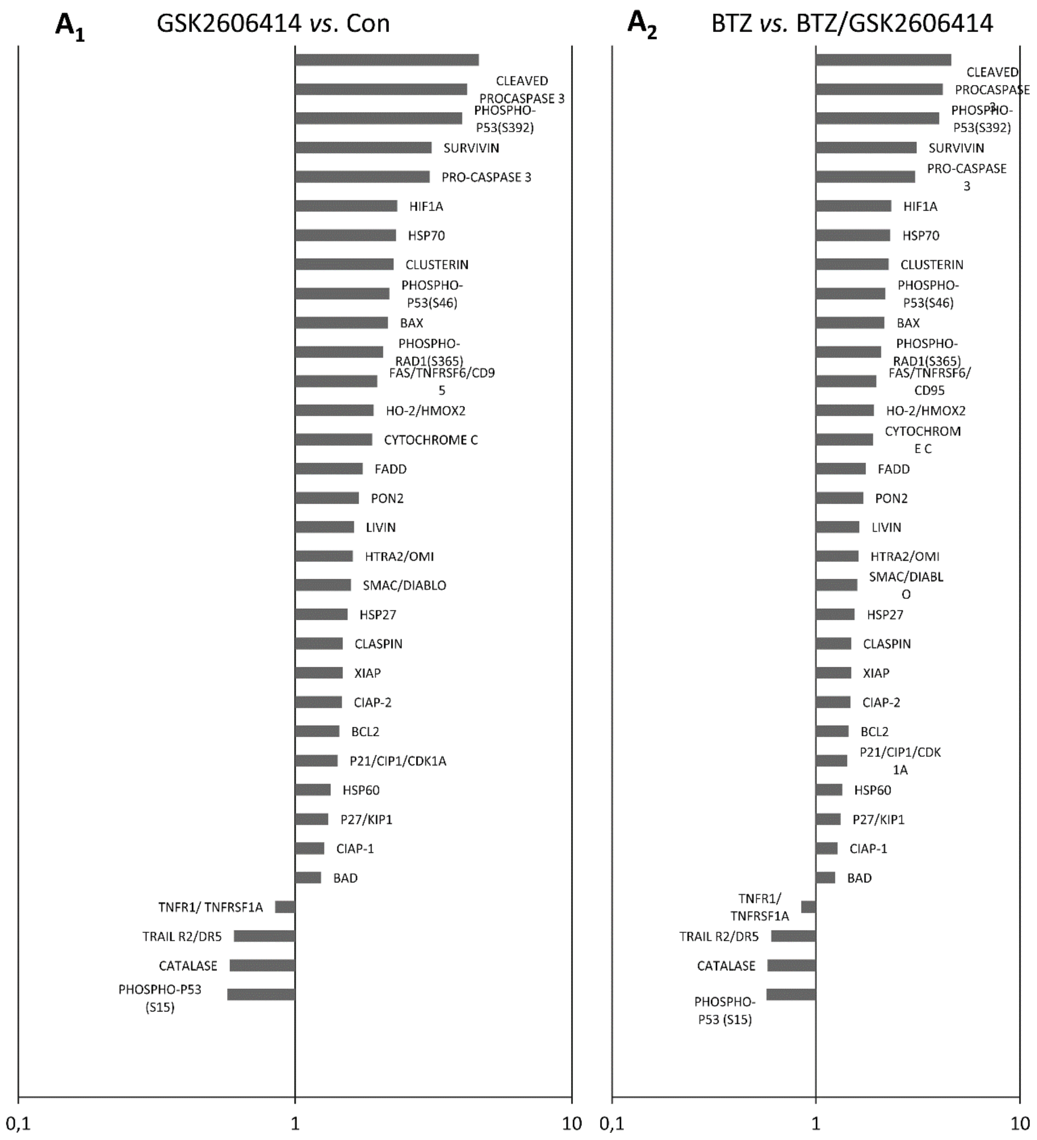

Figure S3. (A1) Relative protein expression in L363 cells of the 35 indicated apoptosis-related proteins in GSK2606414 (10 μM) treated *vs*. untreated (Con) cells **(A2)** Relative protein expression in L363 cells of the 35 indicated apoptosis-related proteins in BTZ (4 nM) *vs*. BTZ (4 nM)/GSK2606414 (10 μM) treated cells.

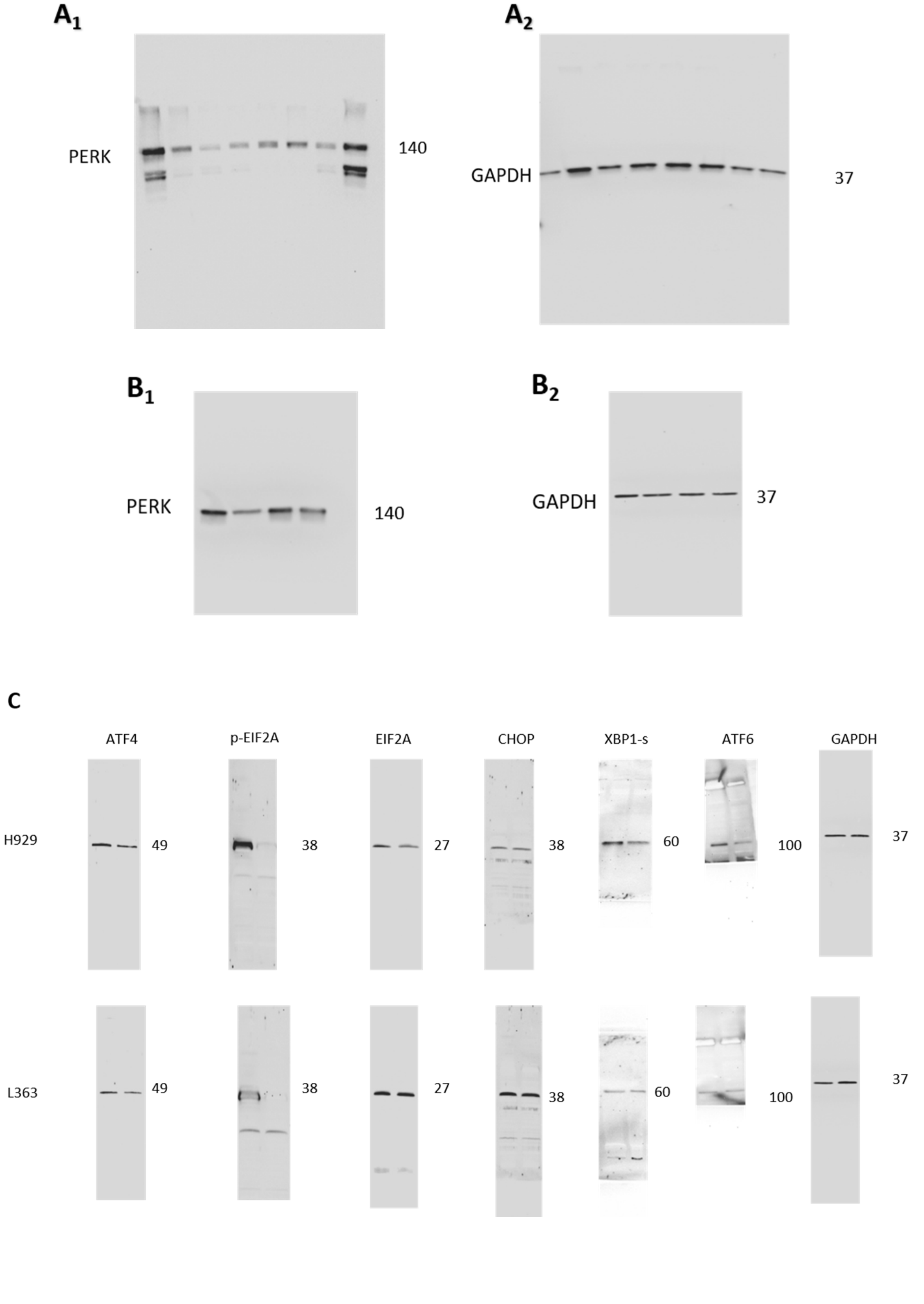
A1
PERK
140
A2
GAPDH
37
B1
PERK
140
B2
GAPDH
37
C
ATF4
p-EIF2A
EIF2A
CHOP
XBP1-s
ATF6
GAPDH
H929
49
38
27
38
60
100
37
L363
49
38
27
38
60
100
37

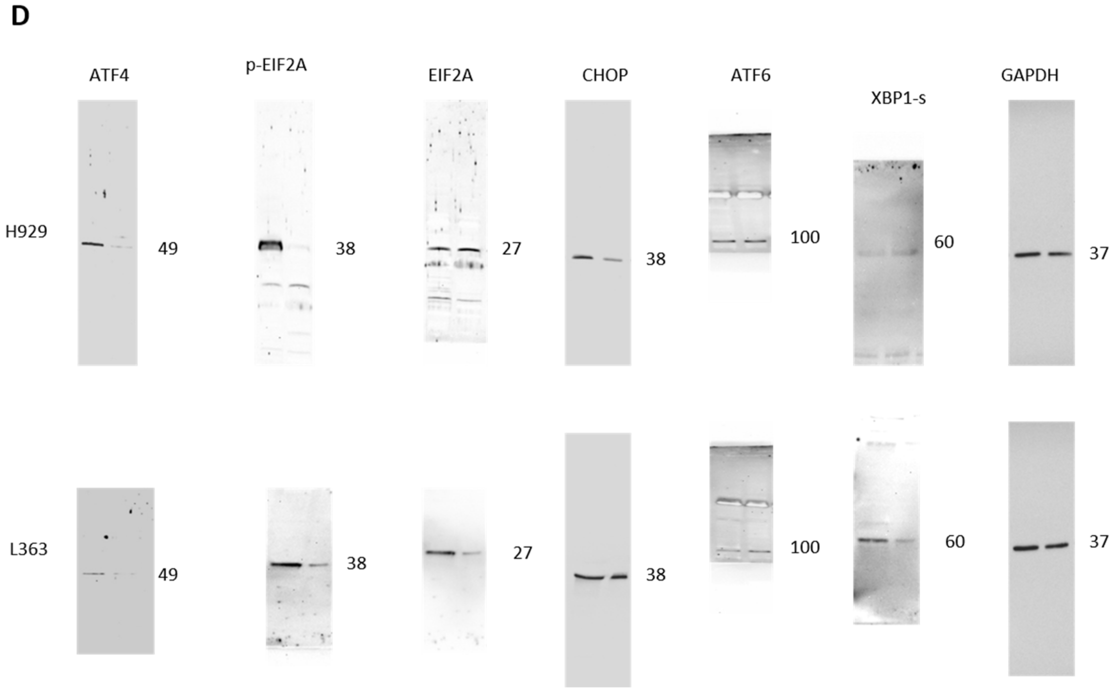
D
ATF4
p-EIF2A
EIF2A
CHOP
ATF6
XBP1-s
GAPDH
H929
49
38
27
38
100
60
37
L363
49
38
27
38
100
60
37

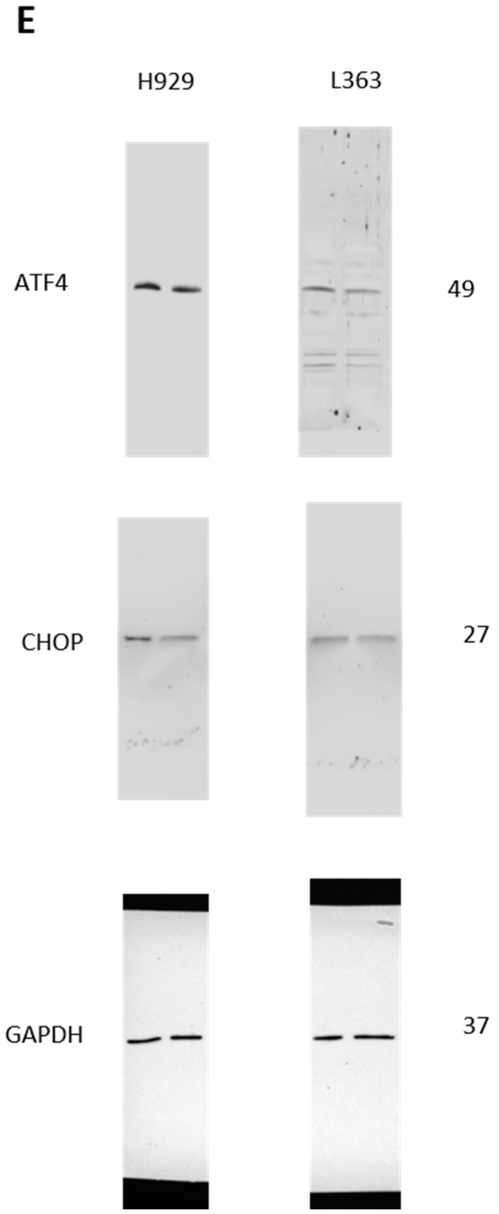

Figure S4. (A1,A2): Uncropped western blot images of Figure 1A2 **(B1,B2)**: Uncropped western blot images of Figure 3B2. **(C)**: Uncropped western blot images of Figure 4A2. **(D)**: Uncropped western blot images of Figure 4B1. **(E)**: Uncropped western blot images of Figure 6C2.

© 2020 by the authors. Licensee MDPI, Basel, Switzerland. This article is an open access article distributed under the terms and conditions of the Creative Commons Attribution (CC BY) license (http://creativecommons.org/licenses/by/4.0/).
